# Association between preoperative hypokalemia and postoperative complications in elderly patients: a retrospective study

**DOI:** 10.1186/s12877-022-03445-1

**Published:** 2022-09-12

**Authors:** Tiantian Chu, Zongfang Wu, Aijun Xu

**Affiliations:** Department of Anesthesiology and Pain Medicine, Tongji Hospital, Huazhong University of Science and Technology, 1095 Jiefang Avenue, Wuhan, 430030 China

**Keywords:** Hypokalemia, The elderly, Risk factors, Complication

## Abstract

**Background:**

Hypokalemia is a common form of electrolyte disorder, which has a higher incidence in hospitalized patients and is closely related to perioperative complications and prognosis. Due to decreased skeletal muscle mass which causes total body potassium reduction, and increased comorbidities, the elderly are more susceptible to hypokalemia.

**Objective:**

To investigate preoperative hypokalemia in elderly patients and its effect on postoperative complications.

**Methods:**

Data were retrospectively collected from the elderly patients who underwent elective surgery from April 2018 to March 2019 and had preoperative blood gas data available. Patients, with age 60 to 100 years, were divided into hypokalemia group (potassium level < 3.5 mmol/L) and normokalemia group (potassium level between 3.5 and 5.5 mmol/L) according to preoperative blood gas analysis. Hypokalemia can be divided into mild (potassium level 3.0 to 3.5 mmol/L), moderate (potassium level 2.5 to 3.0 mmol/L) and severe (potassium level < 2.5 mmol/L), respectively. The risk factors of preoperative hypokalemia and its impact on postoperative complications and prognosis were primary outcomes. Secondary outcomes included postanesthesia care unit (PACU) stay time and hospital length of stay (LOS).

**Results:**

Of 987 participants, 436 (44.17%) developed preoperative hypokalemia, among them 357 (81.88%) mild, 87 (16.74%) moderate and 6 (1.38%) severe. Multivariate logistic regression showed that female gender (OR, 1.851; 95% CI, 1.415–2.421), pre-existing hypokalemia at admission (OR, 4.498; 95% CI, 2.506–8.071), and oral laxative twice or more (OR, 1.823; 95% CI, 1.266–2.624) are risk factors of preoperative hypokalemia. Gynecological and biliopancreatic surgery were more common in hypokalemia group than normokalemia group (*P* <  0.001, *P* <  0.05). There was no significant difference in postoperative complications, PACU stay time, LOS, and 30-day mortality between the two groups (all *P* >  0.05).

**Conclusions:**

Female gender, pre-existing hypokalemia at admission, and oral laxative twice or more are independent risk factors for preoperative hypokalemia in elderly patients. However, postoperative complications and 30-day mortality were not increased, which may be related to monitoring blood gas analysis and prompt correction of potassium levels during surgery.

**Supplementary Information:**

The online version contains supplementary material available at 10.1186/s12877-022-03445-1.

## Introduction

Hypokalemia is a common electrolyte disorder with serum potassium less than 3.5 mmol/L. [1] Its mechanism mainly includes decreased absorption, excessive loss, and abnormal distribution of potassium [2, 3]. Malnutrition, medication use of diuretics, β_2_ receptor agonists or insulin, and preoperative bowel preparation including oral laxatives or enema can lead to the development of hypokalemia [1, 4–7]. Mild hypokalemia with a blood potassium concentration 3.0 mmol/L to 3.5 mmol/L may have no obvious clinical symptoms, however, Mattsson et al. demonstrated that mild hypokalemia increases the risk of stroke and mortality in general population [8]. With a further decrease in blood potassium concentration, moderate and severe hypokalemia may cause serious complications such as muscle weakness, paralytic ileus, metabolic acidosis, urinary retention, rhabdomyolysis, arrhythmia, and even death [1, 4, 9, 10]. In patients with comorbidities, hypokalemia usually portends a worse prognosis [11]. Studies have shown that the prevalence of perioperative hypokalemia was 14 to 40% in general hospital population [1, 4, 12, 13], but evidence on the morbidity and risk factors of preoperative hypokalemia in elderly patients is lacking.

Currently, the number of elderly individuals is increasing. In developing countries, people over 60 years old are classified as the elderly, who have age-related declines in physiology [14, 15]. Due to reduced skeletal muscle mass and increased comorbidities [11, 16], elderly people are more likely to develop hypokalemia and have worse tolerance than young people [4, 17, 18]. A study has shown that perioperative hypokalemia increases the risk of delayed recovery of gastrointestinal function, acute kidney injury, and adverse cardiovascular events [19]. Persistent hypokalemia is also associated with increased all-cause and cardiovascular mortality [20]. Pradeep Arora et al. also demonstrated that preoperative hypokalemia is an independent predictor of mortality and adverse cardiovascular events within 30 days after noncardiac surgery [21]. Thus, closely monitoring and correction of hypokalemia may play an important role in improving the prognosis of patients. Previous studies mainly focused on cardiac surgery [22, 23] or gastrointestinal surgery [5], so it is necessary to analyze the influence of preoperative hypokalemia on postoperative complications and prognosis in elderly patients.

Therefore, the main purpose of this study was to retrospectively analyze preoperative hypokalemia in elderly surgical patients and its impact on postoperative complications and prognosis.

## Methods

### Patients and data collection

Data were collected from elderly patients who underwent elective non-cardiac and non-neurosurgical operations in Tongji Hospital from April 2018 to March 2019. By searching the electronic medical-record system, the information was extracted on age, gender, body mass index (BMI), blood potassium level at admission, and history of diseases including hypertension, arrhythmia, heart failure, chronic obstructive pulmonary disease (COPD) and diabetes. Preoperative diuretics, ACEIs (angiotensin-converting enzyme inhibitors)/ARBs (angiotensin II receptor blockers), oral laxatives, enemas, as well as postoperative complications and LOS were also recorded. The results of blood gas analysis, American Society of Anesthesiologists (ASA) Physical grade, surgical department, time of operation, methods and dosages of intraoperative potassium supplement, PACU stay time and other information were collected from unified Madiston anesthesia system.

Inclusion criteria: (1) age from 60 to 100 years; (2) ASA grade I-III; (3) elective surgery; (4) operation time was 30 min to 6 h. Exclusion criteria: (1) cardiac and neurosurgical surgery; (2) blood potassium data were not obtained after entering the operating room; (3) with pre-existing renal insufficiency, which eGFR (estimated glomerular filtration rate) < 60 mL/min/1.73 m^2^; (4) with a history of Familial hypokalemia.

### Criteria of hypokalemia

Normal serum potassium concentrations range from 3.5 to 5.5 mmol/L. Hypokalemia refers to a serum potassium concentration < 3.5 mmol/L. According to the degree of serum potassium concentration reduction, with serum potassium 3.0 to 3.5 mmol/L, 2.5 to 3.0 mmol/L and < 2.5 mmol/L, hypokalemia can be classified as mild, moderate, and severe respectively [1, 24]. Hyperkalemia refers to a serum potassium concentration > 5.5 mmol/L. [25]

### Outcomes and definitions

The primary outcomes were risk factors of preoperative hypokalemia in elderly surgical patients and its impact on postoperative complications and prognosis. Preoperative hypokalemia was defined as potassium level < 3.5 mmol/L. Potassium was measured by blood gas analysis before anesthesia induction in the operation room. Secondary outcomes included PACU stay time and LOS.

All complications were identified by signs, symptoms, laboratory examination, imaging examination, progress notes, or diagnosis by physician. Pneumonia is diagnosed mainly based on clinical symptoms and lung radiographic changes. Symptomatic evidence includes one of evidence A (fever > 38.0 °C, WBC < 4 × 10^9^ /L or > 12 × 10^9^ /L, altered mental status with no other recognized cause for adults ≥70 years old), and two of evidence B (appearance or increase of purulent sputum, new or worsening cough/dyspnea/tachypnea, rales or bronchial breath sounds, worsening gas exchange). Radiological evidence includes two or more consecutive chest images showing new or persistent infiltration, or cavitation/consolidation [26, 27]. Pleural effusion was mainly determined by chest radiographs. An effusion exceeding 75 mL is often visible on chest radiographs [28]. Fever was defined as postoperative core body temperature > 38.5 °C over 24 hours, excluding clear infectious factors which were listed alone [26]. LOS was defined as the time interval from entering hospital to meeting discharge criteria. 30-day mortality was the percentage of patients who died within 30 days after surgery.

### Data analysis

Patients with blood potassium < 3.5 mmol/L and between 3.5–5.5 mmol/L were included in hypokalemia group and normokalemia group, respectively. The morbidity of hypokalemia was obtained. Preoperative hypokalemia was considered a dependent variable. Independent variables included demographic data, comorbidities (hypertension, arrhythmia, heart failure, COPD, and diabetes), medication use (thiazide/loop/osmotic diuretics, potassium-sparing diuretics, oral laxative, and enema), and surgical types. Variables with significant differences (*P* <  0.05) between two groups were included in logistic regression model. The Omnibus Tests of Model Coefficients (*P* <  0.001) and Hosmer and Lemeshow Test (*P* = 0.357) indicate that the model is generally meaningful and has high goodness of fit. Subsequently, the association of hypokalemia with postoperative outcomes (postoperative complications, PACU stay time, LOS, 30-day mortality) was analyzed. Age and variables which were significant upon univariable analysis, including sex, pre-existing hypokalemia at admission, oral laxative twice or more, and enema twice or more, were included logistic regression model to exclude the influence of confounding factors for postoperative complications and 30-day mortality between two groups.

### Statistical analysis

Statistical Package for Social Sciences (SPSS) software version 21.0 was used for statistical analysis. Continuous variables were expressed as the mean ± standard deviation (SD) or median and interquartile range (IQR), The Shapiro–Wilk test was used to identify the normality of the data distribution. ANOVA or *t*-test was used for normally distributed data, and the Mann-Whitney U test and Kruskal-Wallis test were used for nonparametric test. Categorical variables were described as percentages and analyzed by the Chi-square test or Fisher’s exact test. Multivariate logistic regression models were established to evaluate the risk factors for preoperative hypokalemia and exclude the influence of confounding factors. *P* value < 0.05 was considered statistically significant.

## Results

### Baseline characteristics

**Fig. 1:**
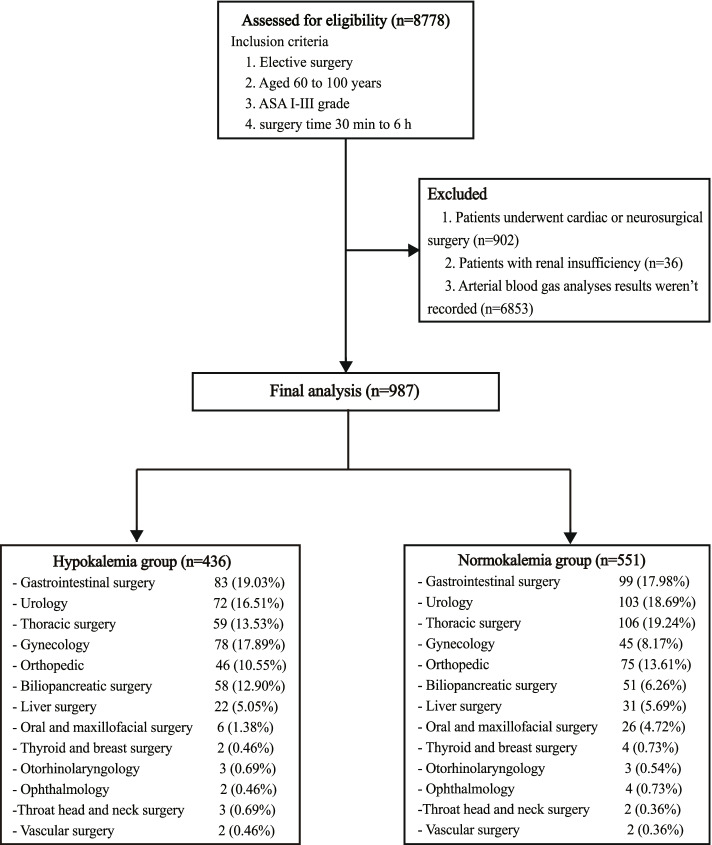
Enrollment flow diagram

A total of 8778 cases were derived from Madiston anesthesia system, among which 987 cases were eligible according to the screening criteria, with 436 in hypokalemia group (44.17%) and 551 in normokalemia group (55.83%). None of patients had preoperative hyperkalemia (potassium level > 5.5 mmol/L). The enrollment flow diagram is shown in Fig. 1.

Demographic characteristics, disease history, and preoperative medication history of all patients were shown in Table 1. There were no significant differences in age, ASA grade, and BMI between the two groups (*P* >  0.05). However, the proportion of female patients was higher in hypokalemia group than normokalemia group (*P* <  0.001). No statistically significant difference between groups was noted in disease history (*P* > 0.05). In terms of preoperative bowel preparation, the proportion of patients with oral laxative or enema twice or more before surgery was significantly higher in hypokalemia group than normokalemia group (*P* <  0.001, *P* <  0.001).

**Table 1:** Characteristics of 987 patients

Characteristics	Hypokalemia (*n* = 436)	Normokalemia (*n* = 551)	*P*	OR	95% CI
Age (years)	66 (63 to 71)	66 (63 to 72)	0.820^^^		
Gender (female)	240 (55.05%)	202 (36.66%)	< 0.001^#^	2.116	1.637–2.733
BMI (kg/m^2^)	23 (21 to 25)	23 (21 to 25)	0.454^^^		
ASA (grade)			0.332^#^		
I	30 (6.88%)	41 (7.44%)			
II	334 (76.61%)	401 (72.60%)			
III	72 (16.51%)	110 (19.96%)			
Hypokalemia (at admission)	54 (12.39%)	16 (2.90%)	< 0.001^#^	4.727	2.665–8.384
Hypertension	181 (41.51%)	200 (36.30%)	0.095^#^	1.246	0.963–1.612
Arrhythmia	18 (4.13%)	29 (5.26%)	0.406^#^	0.775	0.425–1.415
Heart failure	1 (0.23%)	0 (0%)	0.442^+^	0.998	0.993–1.002
COPD	5 (1.15%)	8 (1.45%)	0.676^#^	0.787	0.256–2.424
Diabetes	62 (14.22%)	66 (11.98%)	0.298^#^	1.218	0.840–1.767
Diuretics	16 (3.67%)	22 (3.99%)			
Loop diuretics	3 (0.69%)	7 (1.27%)	0.557^*^	0.538	0.138–2.094
Thiazide diuretics	9 (2.06%)	5 (0.91%)	0.127^#^	2.302	0.766–6.918
Potassium-sparing diuretics	4 (0.92%)	6 (1.09%)	> 0.999^*^	0.841	0.236–2.999
Osmotic diuretics	4 (0.92%)	7 (1.27%)	0.826^*^	0.720	0.209–2.474
ACEIs or ARBs	49 (11.24%)	56 (10.16%)	0.586^#^	1.119	0.746–1.679
Oral laxative (≥2 times)	138 (31.65%)	91 (16.52%)	< 0.001^#^	2.341	1.730–3.167
Enemas (≥2 times)	143 (32.80%)	110 (19.96%)	< 0.001^#^	1.957	1.465–2.613
Time of surgery (min)	135 (100 to 211)	142 (105 to 210)	0.309^^^		
Time of anesthesia (min)	183 (140 to 267)	192 (149 to 259)	0.278^^^		

Data were expressed by median and interquartile range, or frequencies and percentages.

*OR* odds ratio (unadjusted), *CI* confidence interval, *BMI* body mass index, *ASA* American Society of Anesthesiologists, *COPD* chronic obstructive pulmonary disease, *ACEIs* angiotensin-converting enzyme inhibitors, *ARBs* angiotensin II receptor blockers.

^Mann–Whitney U test.

^#^Chi-square test

^*^Corrected Chi-square test

^+^Fisher’s exact test

The distribution of surgical types in two groups was shown in Table 2. Gynecology and biliopancreatic surgery were more common in hypokalemia group than normokalemia group (*P* < 0.001, *P* < 0.05).

**Table 2:** Distribution of surgical types

Surgical types	Hypokalemia (*n* = 436)	Normokalemia (*n* = 551)	*P*	OR	95%CI
Gastrointestinal surgery	83 (19.03%)	99 (17.98%)	0.667^#^	1.074	0.777–1.483
Urology	72 (16.51%)	103 (18.69%)	0.373^#^	0.860	0.618–1.198
Thoracic surgery	59 (13.53%)	106 (19.24%)	0.017^#^	0.657	0.465–0.929
Gynecology	78 (17.89%)	45 (8.17%)	< 0.001^#^	2.450	1.657–3.622
Orthopedics	46 (10.55%)	75 (13.61%)	0.145^#^	0.749	0.506–1.106
Biliopancreatic surgery	58 (13.30%)	51 (9.26%)	0.044^#^	1.504	1.009–2.242
Liver surgery	22 (5.05%)	31 (5.63%)	0.688^#^	0.891	0.508–1.563
Oral and maxillofacial surgery	6 (1.38%)	26 (4.72%)	0.003^#^	0.282	0.115–0.691
Thyroid and breast surgery	2 (0.46%)	4 (0.73%)	0.699^*^	0.630	0.115–3.457
Otorhinolaryngology	3 (0.69%)	3 (0.54%)	> 0.999^*^	1.266	0.254–6.302
Ophthalmology	2 (0.46%)	4 (0.73%)	0.901^*^	0.630	0.115–3.457
Throat head and neck surgery	3 (0.69%)	2 (0.36%)	0.793^*^	1.902	0.316–11.432
Vascular surgery	2 (0.46%)	2 (0.36%)	> 0.999^*^	1.265	0.177–9.017

Data were expressed by frequencies and percentages.

*OR* odds ratio (unadjusted), *CI* confidence interval

^#^Chi-square test

^*^Corrected Chi-square test

### Risk factors of preoperative hypokalemia

A multiple logistic regression equation was established by gender, hypokalemia at admission, oral laxative, laxative enema, and surgical types (Table 3). The risk of preoperative hypokalemia was increased in female patients compared with male patients (*P* < 0.001; OR, 1.851; 95% CI, 1.415–2.421). Pre-existing hypokalemia at admission (*P* < 0.001; OR, 4.498; 95% CI, 2.506–8.071) and oral laxatives twice or more (*P* < 0.001; OR, 1.823; 95% CI, 1.266–2.624) were significantly related to preoperative hypokalemia. Enemas twice or more and surgical types did not increase the risk of preoperative hypokalemia (*P* > 0.05).

**Table 3:** Multivariable logistic regression analysis for risk factors for hypokalemia

Variables	β	SE	Wald	*P*	OR	95% CI
Female gender	0.616	0.137	20.194	< 0.001	1.851	1.415–2.421
Hypokalemia (at admission)	1.504	0.298	25.402	< 0.001	4.498	2.506–8.071
Oral laxatives (≥2 times)	0.600	0.186	10.419	0.001	1.823	1.266–2.624
Enemas (≥2 times)	0.237	0.180	1.733	0.188	1.268	0.890–1.805
Surgical types	0.028	0.035	0.604	0.437	1.028	0.959–1.102

*β* logistic coefficient, *SE* standard error, *OR* odds ratio (adjusted), *CI* confidence interval

### Classification and correction of hypokalemia

According to blood gas analysis results measured before anesthesia induction, the classification of hypokalemia is shown in Table 4. 10% potassium chloride solution and other potassium-containing solutions such as hydroxyethyl starch 130 / 0.4, sodium potassium magnesium calcium and glucose injection were administered intraoperatively to correct hypokalemia. The supplement of potassium in severe hypokalemia patients was higher than that in mild to moderate hypokalemia patients (*P* < 0.001). There was no significant difference in the incidence of postoperative complications among different classifications of hypokalemia.

**Table 4:** Proportion and management of different classifications of hypokalemia

Classification	Mild	Moderate	Severe	Total	*P*
Number (proportion)	357 (81.88%)	73 (16.74%)	6 (1.38%)	436 (100%)	
Supplement of potassium (mmol)	2.01 (0.20 to 4.02)^*^	15.41 (13.40 to 17.42)^*^	16.42 (11.06 to 33.50)		< 0.001^^^
Postoperative complications	29 (8.12%)	9 (12.33%)	1 (16.67%)		0.249^+^

^^^Kruskal Wallis test

^*^P < 0.001 compared with severe hypokalemia

^+^Fisher’s exact test

### Postoperative outcomes

Blood gas analysis was performed in 212 and 233 patients in two groups respectively before they left operating room. Mean blood potassium level was shown in Table 5. There was no statistically significant difference in the incidence of postoperative complications, PACU stay time, LOS, and 30-day mortality between hypokalemia group and normokalemia group (*P* > 0.05). After adjusted by logistic regression, it remained no difference in postoperative complications (*P* > 0.05, OR, 1.047; 95%CI, 0.641–1.709) and 30-day mortality (*P* > 0.05; OR, 0.772; 95%CI, 0.272–2.197). The incidence of various postoperative complications is shown in Table 6.

**Table 5:** Postoperative outcomes

	Hypokalemia	Normokalemia	*P*	*Adjustted P*
Blood potassium level after surgery (mmol/L)	3.50 (3.34 to 3.8)	4.08 (3.8 to 4.40)	< 0.001^^^	
Postoperative complications	39 (8.94%)	41 (7.44%)	0.390^#^	0.854^*^
PACU stay time (min)	32 (22 to 50)	31 (20 to 49)	0.485^^^	
LOS (d)	15 (12 to 21)	16 (13 to 22)	0.091^^^	
30-day mortality	8 (1.83%)	9 (1.63%)	0.809^#^	0.629^*^

Data were expressed by median and IQR, or frequencies and percentages

^*^Adjusted by logistic regression analysis; covariates including age, sex, pre-existing hypokalemia at admission, oral laxative twice or more, and enema twice or more

^Mann–Whitney U test

^#^Chi-square test

**Table 6:** The classification of postoperative complications

Complications	Hypokalemia (*n* = 436)	Normokalemia (*n* = 551)	*P*	OR	95%CI
Pulmonary	14 (3.21%)	11 (2.00%)	0.308	1.629	0.732–3.624
Pneumonia	5 (1.15%)	4 (0.73%)			
Pleural effusion	5 (1.15%)	3 (0.54%)			
Atelectasis	3 (0.69%)	2 (0.36%)			
Else	1 (0.23%)	2 (0.36%)			
Gastrointestinal	4 (0.92%)	7 (1.27%)	0.763	0.720	0.209–2.474
Nausea and vomiting	3 (0.69%)	5 (0.91%)			
Diarrhea	1 (0.23%)	2 (0.36%)			
Cardiovascular	2 (0.46%)	4 (0.73%)	0.699	0.630	0.115–3.457
Arrhythmia	1 (0.23%)	2 (0.36%)			
Cardiac arrest	1 (0.23%)	2 (0.36%)			
Neuropsychiatric	3 (0.69%)	1 (0.18%)	0.327	3.811	0.395–36.762
Wound	2 (0.46%)	9 (1.63%)	0.125	0.278	0.060–1.291
Bleeding	0 (0%)	3 (0.54%)			
Poor healing	1 (0.23%)	4 (0.73%)			
Else	1 (0.23%)	2 (0.36%)			
Infection	7 (1.61%)	3 (0.54%)	0.117	2.981	0.766–11.594
Thrombosis	2 (0.46%)	5 (0.91%)	0.474	0.503	0.097–2.606
Fever	5 (1.15%)	1 (0.18%)	0.093	6.381	0.743–54.816

*OR* odds ratio, *CI* confidence interval

Data were expressed by frequencies and percentages

Chi-square test

## Discussion

In this study, female gender, pre-existing hypokalemia at admission, and oral laxative twice or more before surgery were independent risk factors for preoperative hypokalemia. There was no significant difference in the incidence of postoperative complications and 30-day mortality between groups.

Alfonzo et al. found that there was no significant difference in the prevalence of hypokalemia between males and females in hospitalized patients [10]. However, in our study, female gender was an independent risk factor for preoperative hypokalemia in elderly patients. Kleinfeld et al. showed that increasing incidence of hypokalemia in female patients mainly occurred in the elderly population, which is similar to our study. It may be related to more significant decline of muscle mass in elderly women, resulting in a decrease in potassium available for intracellular and extracellular exchange in whole body [29]. Moreover, because females undergoing gynecological surgery often require bowel preparation, whether hypokalemia in female gender is related to types of surgery remains to be explored.

Our study showed that pre-existing hypokalemia at admission is one of the risk factors of preoperative hypokalemia. Bardak et al. reported that the incidence of community-acquired hypokalemia in elderly patients was 3.24%, and LOS, hospital cost, and all-cause mortality in hypokalemia group were higher [11]. Of note, a total of 7.09% of all patients in our study had hypokalemia at admission, more than twice the rate reported by Bardak et al. The discrepancy may be attributed to dietary structure and metabolic characteristics [30] of different ethnic groups. Du et al. have shown that potassium intake is below half of the recommended amount in China [31, 32]. Also, ethnic differences in pharmacokinetics or pharmacodynamics can affect the absorption, metabolism, and excretion of drugs [33, 34]. These studies seem to support our hypothesis, but there is still a lack of relevant studies to explore differences in hypokalemia among ethnic groups.

Bowel preparation is often implemented before gastrointestinal surgery. Preoperative oral laxative and enema are the main methods, which cause transient watery diarrhea and loss of a large amount of digestive juice, resulting in a decline in blood potassium, and patients are prone to preoperative hypokalemia [1, 5]. Our study found that two or more oral laxatives increased the risk of preoperative hypokalemia, whereas enemas did not. The results suggested that oral laxative may have a greater impact on blood potassium levels than enema. Sadaba et al. also found that sodium phosphate enema did not affect blood potassium in healthy volunteers [35], which confirmed that enema has a limited effect on blood potassium.

Ben and Nilsson et al. have shown that diuretics such as thiazides can develop into hypokalemia by increasing the excretion of potassium [6, 12]. We investigated the association of loop/thiazide/osmotic diuretics with preoperative hypokalemia, however, the results were not positive. Potassium-elevating medications such as ACEis/ARBs [36] also did not have a significant effect on potassium levels and no preoperative hyperkalemia was observed in our study. These results may be due to the exclusion of patients with renal insufficiency, lower frequency of use, and concomitant use of counteracting drugs. Similar results also exist in researches of Hirai [37] and Zhu [38]. This is likely to indicate that preoperative potassium disturbances in elderly hospitalized patients with normal renal function are more manifested as hypokalemia. Other drugs such as Yokukansan [37], sulfamethoxazole/trimethoprim [25], and licorice [39] also cause blood potassium disorder by affecting excretion. However, due to the low proportion of patients taking these drugs, it was difficult to compare their effects on preoperative hypokalemia. Furthermore, it is well known that potassium is mainly excreted through kidneys, and renal dysfunction often leads to electrolyte disturbances, including hypokalemia and hyperkalemia. To investigate risk factors for preoperative hypokalemia in elderly hospitalized patients other than renal causes, this study excluded patients with pre-existing renal dysfunction, and future studies will further investigate the preoperative hypokalemia in this population.

Kardalas et al. have mentioned that symptoms of hypokalemia can vary from absent to muscle weakness, reduced gastrointestinal motility, arrhythmias, heart failure, etc., which are related to the severity and duration of hypokalemia [40]. Ebrahim et al. reported that preoperative hypokalemia increased 30-day mortality in elderly patients with open abdominal surgery, especially emergency surgery [41]. However, preoperative hypokalemia did not affect postoperative complications and 30-day mortality in our study. One reason may be that most patients underwent elective surgery and the surgical types were endoscopic. And most of the hypokalemia observed was mild in our study. Furthermore, since patients with preoperative hypokalemia were treated with intravenous potassium supplementation during the operation, blood gas analysis showed blood potassium in both groups were within normal range at the time of leaving operating room. Perhaps due to mild hypokalemia and timely intraoperative correction of blood potassium, postoperative complications did not increase in our study.

There were several limitations in our study. First, preoperative blood potassium is mainly obtained by blood gas analysis in our study. The comparison of potassium measured by blood gas analyzers and laboratory automated analyzers was not available due to retrospective nature. Future prospective studies may be conducted to confirm the consistency between the two methods. Second, the types of surgeries included in this study are extensive and heterogeneous, and no stratified discussion has been conducted. Therefore, patients from a specific surgery, such as gynecology, biliopancreatic surgery, etc. can be selected for follow-up research. Third, the incidence of preoperative hypokalemia should be interpreted carefully because the inclusion depended on the availability of blood gas data, which may lead to potential selection bias. Future prospective studies need to consider the consistency of clinical characteristics of the included patients.

## Conclusions

Our study indicated that female gender, pre-existing hypokalemia at admission, and oral laxative twice or more are independent risk factors for elderly patients. Gynecology and biliopancreatic surgery are also associated with preoperative hypokalemia. No difference in the incidence of postoperative complications may be related to mild hypokalemia and timely correction of intraoperative potassium levels. Therefore, it is recommended to routinely monitor perioperative blood potassium for elderly patients in future work, especially for those with risk factors. Departments such as gynecology and biliopancreatic surgery should strengthen preoperative management of high risk elderly patients.

## Supplementary Information

**Additional file 1.**

## Data Availability

All data generated during this study are included in this published article and its Supplementary information files. Available upon request from corresponding author at ajxu@tjh.tjmu.edu.cn.

## Abbreviations

ACEIs: Angiotensin-converting enzyme inhibitors
ARBs: Angiotensin II receptor blockers
ASA: American Society of Anesthesiologists
BMI: Body mass index
CI: Confidence interval
COPD: Chronic obstructive pulmonary disease
GFR: Glomerular filtration rate
IQR: Interquartile range
LOS: Length of stay
OR: Odds ratio
PACU: Postanesthesia care unit
PPC: Postoperative pulmonary complications
SD: Standard deviation
SPSS: Statistical Package for Social Sciences
WBC: White blood cell

## References

[1] Krogager ML, Kragholm K, Thomassen JQ, Sogaard P, Lewis BS, Wassmann S, Baumgartner I, Ceconi C, Schmidt TA, Kaski JC (2021). Update on management of hypokalaemia and goals for the lower potassium level in patients with cardiovascular disease: a review in collaboration with the European Society of Cardiology Working Group on cardiovascular pharmacotherapy. Eur Heart J Cardiovasc Pharmacother.

[2] Salim L (2007). Approach to Hypokalemia. Acta Med Indones.

[3] Viera AJ, Wouk N (2015). Potassium Disorders: Hypokalemia and Hyperkalemia. Am Fam Physician.

[4] Greco A, Rabito G, Pironi M, Bissig M, Parlato S, Andreocchi L, Bianchi G, Poretti Guigli M, Llamas M, Monotti R (2016). Hypokalaemia in hospitalised patients. Swiss Med Wkly.

[5] Ho JM, Juurlink DN, Cavalcanti RB (2010). Hypokalemia following polyethylene glycol-based bowel preparation for colonoscopy in older hospitalized patients with significant comorbidities. Ann Pharmacother.

[6] Ben SC, Hmouda H, Bouraoui K (2009). Drug-induced hypokalaemia. Curr Drug Saf.

[7] Khan S, Khan SU (2020). Adverse drug event of hypokalaemia-induced cardiotoxicity secondary to the use of laxatives: a systematic review of case reports. J Clin Pharm Ther.

[8] Mattsson N, Nielsen OW, Johnson L, Prescott E, Schnohr P, Jensen GB, Kober L, Sajadieh A (2018). Prognostic impact of mild hypokalemia in terms of death and stroke in the general population-a prospective population study. Am J Med.

[9] Schaefer TJ, Wolford RW (2005). Disorders of potassium. Emerg Med Clin North Am.

[10] Alfonzo AV, Isles C, Geddes C, Deighan C (2006). Potassium disorders--clinical spectrum and emergency management. Resuscitation.

[11] Bardak S, Turgutalp K, Koyuncu MB, Hari H, Helvaci I, Ovla D, Horoz M, Demir S, Kiykim A (2017). Community-acquired hypokalemia in elderly patients: related factors and clinical outcomes. Int Urol Nephrol.

[12] Nilsson E, Gasparini A, Arnlov J, Xu H, Henriksson KM, Coresh J, Grams ME, Carrero JJ (2017). Incidence and determinants of hyperkalemia and hypokalemia in a large healthcare system. Int J Cardiol.

[13] Lippi G, Favaloro EJ, Montagnana M, Guidi GC (2010). Prevalence of hypokalaemia: the experience of a large academic hospital. Intern Med J.

[14] Alvis BD, Hughes CG (2015). Physiology considerations in geriatric patients. Anesthesiol Clin.

[15] Islas-Granillo H, Medina-Solis CE, de Lourdes M-CM, de la Rosa-Santillana R, Fernandez-Barrera MA, Villalobos-Rodelo JJ, Hernandez-Martinez CT, de Jesus N-HJ, Mendoza-Rodriguez M (2018). Prevalence of multimorbidity in subjects aged >/=60 years in a developing country. Clin Interv Aging.

[16] Kojima T, Mizokami F, Akishita M (2020). Geriatric management of older patients with multimorbidity. Geriatr Gerontol Int.

[17] Abensur VL, Ferreira JP, Asseray N, Trombert-Paviot B, Montassier E, Legrand M, Girerd N, Boivin JM, Chouihed T, Rossignol P (2020). Hypokalemia is frequent and has prognostic implications in stable patients attending the emergency department. PLoS One.

[18] Steen B (1981). Hypokalemia--clinical spectrum and etiology. Acta Med Scand Suppl.

[19] Lu G, Xu L, Zhong Y, Shi P, Shen X (2014). Significance of serum potassium level monitoring during the course of post-operative rehabilitation in patients with hypokalemia. World J Surg.

[20] Krogager ML, Sogaard P, Torp-Pedersen C, Boggild H, Lee CJ, Bonde A, Thomassen JQ, Gislason G, Pareek M, Kragholm K (2020). Impact of plasma potassium normalization on short-term mortality in patients with hypertension and hypokalemia or low normal potassium. BMC Cardiovasc Disord.

[21] Arora P, Pourafkari L, Visnjevac O, Anand EJ, Porhomayon J, Nader ND (2017). Preoperative serum potassium predicts the clinical outcome after non-cardiac surgery. Clin Chem Lab Med.

[22] Sanjay O (2004). Pre-operative serum potassium levels and peri-operative outcomes in patients undergoing cardiac surgery. Indian J Clin Biochem.

[23] Wahr JA, Parks R, Boisvert D, Comunale M, Fabian J, Ramsay J, Mangano DT (1999). Preoperative serum potassium levels and perioperative outcomes in cardic surgery patients. Multicenter Study of Perioperative Ischemia Research Group. JAMA.

[24] Peng H, Zhang Q, Qian J, Ruan F, Mai H, Wang Z, Liu M, Wang Z, Chen H, Li J (2020). Electrolyte disorders are ERAS-associated in patients undergoing hepato-pancreato-biliary surgery. Langenbeck's Arch Surg.

[25] Hirai T, Yamaga R, Ishikawa Y, Hanada K, Iwamoto T, Itoh T (2021). Effect of high-dose sulfamethoxazole/trimethoprim and glucocorticoid use on hyperkalemic event: a retrospective observational study. J Infect Chemother.

[26] Barnes J, Hunter J, Harris S, Shankar-Hari M, Diouf E, Jammer I, Kalkman C, Klein AA, Corcoran T, Dieleman S (2019). Systematic review and consensus definitions for the standardised endpoints in perioperative medicine (StEP) initiative: infection and sepsis. Br J Anaesth.

[27] Horan TC, Andrus M, Dudeck MA (2008). CDC/NHSN surveillance definition of health care-associated infection and criteria for specific types of infections in the acute care setting. Am J Infect Control.

[28] Sancho JF, Blasco H, de Pablo Gafas A, FRP EPR, Candeira SR, Velázquez ÁS, Cuadrado LV, Victoria Villena Garrido (2006). Diagnosis and Treatment of Pleural Effusion. Arch Bronconeumol.

[29] Kleinfeld M, Borra S, Gavani S, Corcoran A (1993). Hypokalemia: are elderly females more vulnerable?. J Natl Med Assoc.

[30] Turban S, Miller ER, Ange B, Appel LJ (2008). Racial differences in urinary potassium excretion. J Am Soc Nephrol.

[31] Du S, Wang H, Zhang B, Popkin BM (2020). Dietary potassium intake remains low and sodium intake remains high, and Most sodium is derived from home food preparation for Chinese adults, 1991-2015 trends. J Nutr.

[32] Du S, Batis C, Wang H, Zhang B, Zhang J, Popkin BM (2014). Understanding the patterns and trends of sodium intake, potassium intake, and sodium to potassium ratio and their effect on hypertension in China. Am J Clin Nutr.

[33] Johnson JA (1997). Influence of race or ethnicity on pharmacokinetics of drugs. Pharm Sci.

[34] Chen M (2006). Ethnic or racial differences revisited: impact of dosage regimen and dosage form on pharmacokinetics and pharmacodynamics. Clin Pharmacokinet.

[35] Sédaba B, Azanza JR, Campanero MA, Garcia-Quetglas E, Muñoz MJ, Marco S (2006). Effects of a 250-mL enema containing sodium phosphate on electrolyte concentrations in healthy volunteers: an open-label, randomized, controlled, two-period, crossover clinical trial. Curr Ther Res Clin Exp.

[36] Ben Salem C, Badreddine A, Fathallah N, Slim R, Hmouda H (2014). Drug-induced hyperkalemia. Drug Saf.

[37] Hirai T, Yamaga R, Kei M, Hosohata K, T. (2020). I: geriatric patients are at a high risk of hypokalemia associated with Yokukansan preparation: a retrospective cohort study. Biol Pharm Bull.

[38] Zhu Q, Li X, Tan F, Deng Y, Gong C, Hu J, Huang P, Zhou S (2018). Prevalence and risk factors for hypokalemia in patients scheduled for laparoscopic colorectal resection and its association with post-operative recovery. BMC Gastroenterol.

[39] Awad N, Makar G, Burroughs V, Ravi P, Burroughs SR (2020). Licorice-induced apparent mineralocorticoid excess causing persistent hypertension and hypokalemia. Acta Endocrinol (Buchar).

[40] Kardalas E, Paschou SA, Anagnostis P, Muscogiuri G, Siasos G, Vryonidou A (2018). Hypokalemia: a clinical update. Endocr Connect.

[41] Ebrahim M, Larsen PB, Hannani D, Liest S, Jørgensen LN, Jørgensen HL. Preoperative risk factors including serum levels of potassium, sodium, and creatinine for early mortality after open abdominal surgery: a retrospective cohort study. BMC Surg. 2021;21(1). 10.1186/s12893-021-01070-0. 10.1186/s12893-021-01070-0 PMC7836189 33499844

